# Environmental pressures, tumor characteristics, and death rate in a female breast cancer cohort: a seven-years Bayesian survival analysis using cancer registry data from a contaminated area in Italy

**DOI:** 10.3389/fpubh.2023.1310823

**Published:** 2024-01-08

**Authors:** Orazio Valerio Giannico, Simona Carone, Margherita Tanzarella, Claudia Galluzzo, Antonella Bruni, Giovanna Maria Lagravinese, Ivan Rashid, Lucia Bisceglia, Rodolfo Sardone, Francesco Addabbo, Sante Minerba, Antonia Mincuzzi

**Affiliations:** ^1^Unit of Statistics and Epidemiology, Local Health Authority of Taranto, Taranto, Italy; ^2^Coordination Center of the Apulia Cancer Registry, Strategic Regional Agency for Health and Social Care of Apulia, Bari, Italy; ^3^Healthcare Management, Local Health Authority of Taranto, Taranto, Italy

**Keywords:** breast cancer, female breast cancer, cancer survival, environmental contamination, environmental pollution, cancer epidemiology

## Abstract

**Introduction:**

In Taranto, Southern Italy, adverse impacts on the environment and human health due to industrial installations have been studied. In the literature, few associations have been reported between environmental factors and breast cancer mortality in women. The aim of this study was to investigate the relationships between residence in areas with high environmental pressures, female breast cancer characteristics, and death rate.

**Methods:**

Data from the Taranto Cancer Registry were used, including all women with invasive breast cancer diagnosed between 01 January 2015 and 31 December 2020 and with follow-up to 31 December 2021. Bayesian mixed effects logistic and Cox regression models were fitted with the approach of integrated nested Laplace approximation, adjusting for patients and disease characteristics.

**Results:**

A total of 10,445 person-years were observed. Variables associated with higher death rate were residence in the contaminated site of national interest (SIN) (HR 1.22, 95% CrI 1.01–1.48), pathological/clinical stage III (HR 2.77, 95% CrI 1.93–3.97) and IV (HR 17.05, 95% CrI 11.94–24.34), histological grade 3 (HR 2.50, 95% CrI 1.20–5.23), Ki-67 proliferation index of 21–50% (HR 1.42, 95% CrI 1.10–1.83) and > 50% (HR 1.81, 95% CrI 1.29–2.55), and bilateral localization (HR 1.65, 95% CrI 1.01–2.68). Variables associated with lower death rate were estrogen and/or progesterone receptor positivity (HR 0.61, 95% CrI 0.45–0.81) and HER2/neu oncogene positivity (HR 0.59, 95% CrI 0.44–0.79).

**Discussion:**

The findings confirmed the independent prognostic values of different female breast cancer characteristics. Even after adjusting for patients and disease characteristics, residence in the SIN of Taranto appeared to be associated with an increased death rate.

## Introduction

Female breast cancer is a leading cause of cancer incidence and mortality worldwide, with a global estimate of 2.3 million incident cases, 7.8 million prevalent cases, and 685,000 deaths in 2020. It is a global phenomenon affecting individuals of any age after puberty. Among all types of cancer, it causes the most disability-adjusted life years (DALYs) among women (1, 2).

Early cancer detection (screening) and treatment (surgery, radiation, and medical therapy) have proven to be effective, achieving survival probabilities up to 90% or higher (1). Specifically, the treatment of breast cancer is based on the tumor’s biological subtyping. Numerous disease characteristics, such as TNM staging, histological grading, proliferation activity (Ki-67%), estrogen receptor (ER), progesterone receptor (PR) and HER2/neu positivity, topography, and bilaterality, have been researched as potential prognostic indicators or as targets for specific therapies (1, 3–7). Tumor, node, and metastases (TNM) classification is a system used to describe the amount and spread of cancer in a patient’s body: T describes the size of the tumor and any spread of cancer into nearby tissues, N describes the spread of cancer to nearby lymph nodes, and M describes metastasis, i.e., the spread of cancer to other parts of the body. TNM combinations are grouped into five less-detailed stages, from 0 (carcinoma *in situ*: abnormal cells are present but have not spread to nearby tissues) to I-II-III (invasive cancer: the higher the number, the larger the tumor and the more it has spread into nearby tissues) to IV (invasive, metastatic cancer: cancer has spread to distant parts of the body) (3, 8–10). In addition to TNM staging, histologic grading from 1 to 3 is an important predictor of disease outcome in breast cancer patients, with a higher tumor grade (lower differentiation) being associated with poorer prognosis (differentiation describes how much a tumor resembles the normal tissue from which it arose) (3, 5, 11). Related to grading is the expression of Ki-67, a nuclear protein present during the late G1, S, G2, and M phases of the cell cycle. It has been demonstrated that high Ki-67 expression (%), which reflects the proliferation activity of tumor cells, is associated with a higher risk of relapse and worse survival in breast cancer patients (3, 5). Hormonal receptor status is another important prognostic factor in breast cancer patients. Estrogen receptor-positive (ER+) and progesterone receptor-positive (PR+) tumors are likely to respond to endocrine therapies such as tamoxifen or aromatase inhibitors. Hormone therapy reduces the chance of recurrence by nearly half. Moreover, breast cancers may independently overexpress a molecule called the HER2/neu oncogene and these HER2/neu + tumors are amenable to treatment with targeted biological agents such as trastuzumab, with improvement of survival (1, 3, 4). Moreover, associations were reported between cancer topography, laterality, and death rate. Specifically, better survival was associated with greater tumor-nipple distance in old patients, while an increased death rate was reported for synchronous bilateral breast cancer patients (6, 7).

With regard to socio-economic and environmental factors, a shorter breast cancer-specific survival in women from disadvantaged neighborhoods has been reported (12). Evidence from a meta-analysis indicated that patients residing in rural areas were more likely to be diagnosed with more advanced breast cancer compared with patients from urban areas (13). Moreover, associations were reported between air pollutants and mortality in women with breast cancer (14, 15).

In some areas of the province of Taranto, a coastal city in the Apulia Region, Southern Italy, various industrial installations and polluting sources (a steel plant, an oil refinery, urban discharges, harbor activities, and the ship-yard of the Italian Navy) have been operating in close proximity to the resident population for decades with well-known and extensively studied adverse impacts on the environment and human health (16–30). With regard to environmental, feed, and food impacts, it is of particular importance that the area shows contamination of these matrices by metals and persistent organic pollutants, specifically dioxins and PCBs. Moreover, some of these substances have been detected in human biological samples (17, 20–25). As far as human health effects are concerned, evidence has been produced after studying the populations who resided in the contaminated site of national interest (SIN) of Taranto. In particular, cohort studies have reported an increased risk for different types of cancer incidence, including breast cancer incidence in women (16, 27). Some studies have also noted an increased risk for all-cause hospitalization; for circulatory, respiratory, digestive, and urinary diseases hospitalization; and for different types of cancer hospitalization, including breast cancer hospitalization in women (16, 29, 30). Different studies have also indicated an increased risk for all-cause mortality; for circulatory and digestive diseases mortality; and for some types of cancer mortality, with no significant evidence for breast cancer mortality in women (16, 19, 26, 29, 30).

To summarize, associations have been reported between the aforementioned factors and breast cancer mortality in women. The aim of this study was to investigate the relationships between residence in areas with high environmental pressures, female breast cancer characteristics, and death rate.

## Methods

### Study area and baseline epidemiological data

**Figure 1:**
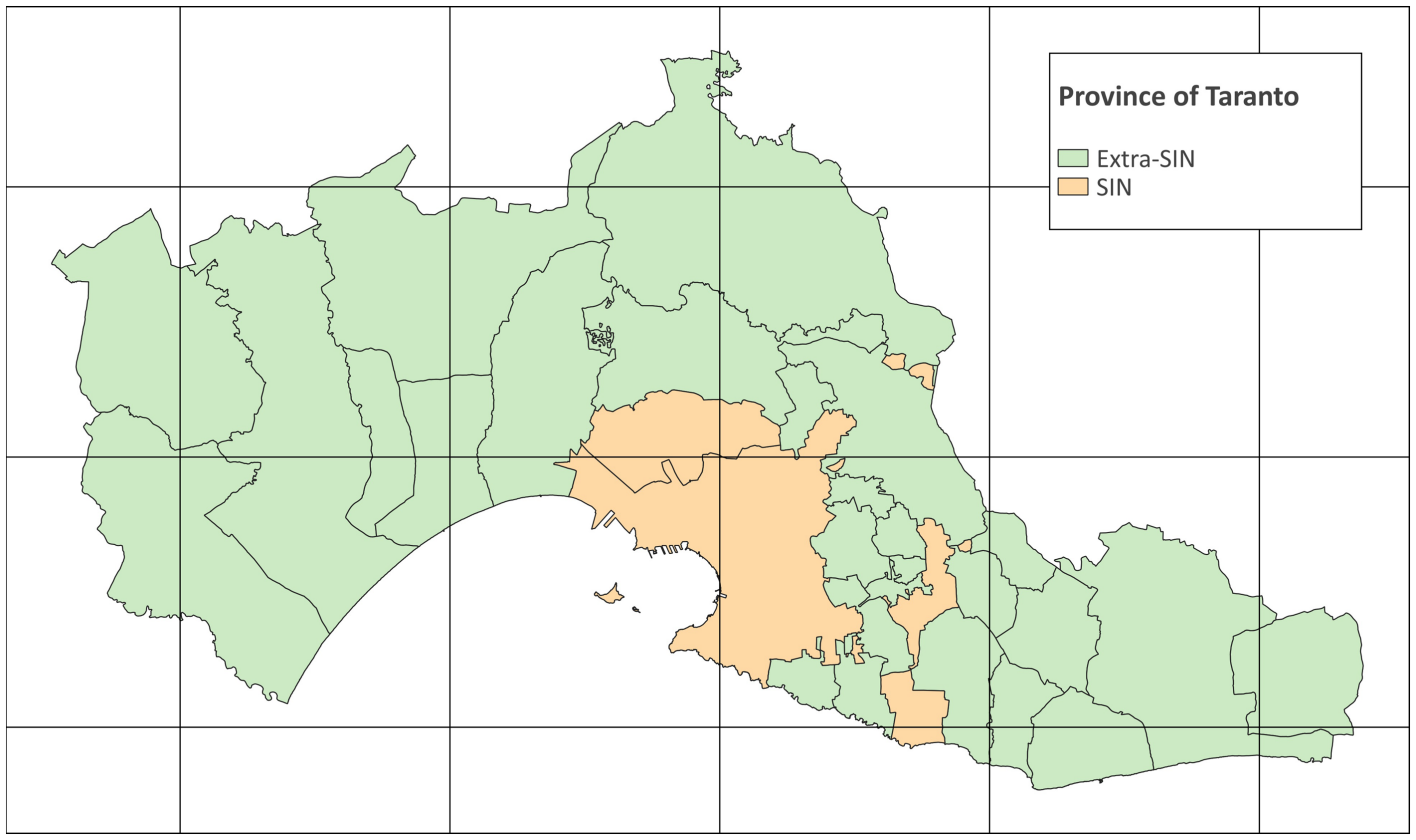
Map of the province of Taranto (grid interval: 20 km) (EPSG:32632 – WGS 84 / UTM zone 32N) (Modified from Italian National Institute of Statistics. Administrative boundaries. https://www.istat.it/it/archivio/222527).

The study area is the province of Taranto, which consists of 29 municipalities with a total resident population of 559,892 inhabitants on 1 January 2022 (31). The SIN of Taranto consists of two municipalities, Taranto (the provincial capital) and Statte, with 189,461 and 13,136 inhabitants, respectively, on 1 January 2022 (29–31). The study area with municipalities and SIN is shown in Figure 1. The map was created with QGIS version 3.28.4.

From 2015 to 2019, Taranto Province recorded 2,446 female breast cancer (ICD10 codes C50.0 to C50.9) cases, with a directly standardized rate of 147.6 cases per 100,000 inhabitants and a median age of 61 years. In the same period, 63% of patients with breast cancer requiring hospitalization were admitted to a hospital in the Taranto Province, 25% to an extra-provincial hospital in the Apulia Region, and 12% to an extra-regional hospital. Between 2013 and 2017, the relative standardized 5-year female breast cancer survival was 85.6 (95% CI 83.1–87.7) (27).

From 2013 to 2017, in the SIN, 216 female breast cancer deaths were recorded, with a standardized mortality ratio (reference: Apulia Region) of 99 (90% CI 89–111) (29). From 2015 to 2019, in the SIN, 979 female breast cancer cases were recorded, with a directly standardized rate of 155.6 cases per 100,000 inhabitants and a standardized incidence ratio (reference: Taranto Province) of 109.3 (95% CI 102.5–116.3) (27).

### Data source and cancer cohort

Data collected from the Taranto Cancer Registry of the Italian Association of Cancer Registries (AIRTUM) were used, including all women with invasive breast cancer (ICD10 codes C50.0 to C50.9) diagnosed between 1 January 2015 and 31 December 2020 who resided in Taranto Province at the time of diagnosis. The follow-up period considered for this study was until 31 December 2021. Death certificate only cases (*n* = 29), cases registered based on an autopsy report (*n* = 1), and patients under 30 years (*n* = 12) were excluded. As a general rule, baseline patients and disease characteristics refer to the time of diagnosis. Mortality data (all-cause mortality) relative to the follow-up period (2015–2021) were retrieved from the Taranto Province’s Causes of Death and Health Registries. Patients with no mortality follow-up information due to extra-provincial transfer before 31 December 2021 (right-censoring, loss-to-follow-up) contributed to the person-time until the date of transfer (*n* = 7).

### Study design and variables

This is a retrospective individual observational study with different regression analyses carried out cross-sectionally (prevalence study) and longitudinally (incidence study, survival analysis). Residence in the areas with high environmental pressures (SIN) was used as an environmental exposure proxy. Tumor characteristics at the time of diagnosis were pathological/clinical staging (TNM I to IV), histological grading (grade 1 to 3), proliferation index (Ki-67%), estrogen receptor (ER) and/or progesterone receptor (PR) status (positivity cut-off is ≥1% of positive cells), epidermal receptor (HER2/neu) status (immunohistochemistry, IHC; fluorescence *in situ* hybridization, FISH), topography (ICDO3T classification. C50.0–1: nipple and areola, central portion of breast. C50.2–5: upper-inner/lower-inner/upper-outer/lower-outer quadrant of breast. C50.6–8: axillary tail of breast, overlapping lesion of breast), and laterality (unilateral right, unilateral left, and synchronous bilateral breast cancer) (3).

In the cross-sectional study, the studied outcomes were each of the tumor characteristics (prevalence), and the studied exposure was residence in areas with high environmental pressures. The aim of this step was to assess possible associations between environmental factors and each of the tumor characteristics. In the longitudinal study, the studied outcome was all-cause death (incidence), and the studied exposures were residence in areas with high environmental pressures and tumor characteristics. The aim of this step was to assess possible associations between environmental factors, tumor characteristics, and death. Adjustment variables recorded at the time of diagnosis were age class (30–39, 40–49, 50–59, 60–69, 70–79, and ≥ 80 years), year, month, patient ID, municipality of residence, and tumor morphology (ICDO3M classification). Adjustment for the patient’s ID and municipality of residence was provided to account for the heterogeneity related to possible unobserved individual or ecological level variables (e.g., genetics, heredity, tobacco use, alcohol consumption, deprivation index, and access to health services).

### Statistical analysis

Data analysis was performed using R version 4.2.3. Bayesian inference was performed with package INLA version 22.12.16. Complex models could be fitted with the Bayesian approach, including generalized linear models and survival analyses. The possible non-independence and heterogeneity of observations could be taken into account by fitting mixed models with both fixed and random effects. While traditional survival analysis relies on parameter estimation based on partial likelihood, Bayesian approaches for time-to-event data allow us to use the full likelihood to estimate all unknown elements in the model. Bayesian generalized linear models comprise Bayesian logistic regression for binary response data. However, the computation of the posterior and the other quantities of interest in these complex models is usually much more difficult than frequentist calculations. The Integrated Nested Laplace Approximation (INLA) is a deterministic method for Bayesian calculations that applies to a wide class of models called Latent Gaussian Models. INLA provides fast and accurate approximations to the posterior marginals through a clever use of Laplace approximations and advanced numerical methods taking computational advantage of sparse matrices. In most cases, INLA is both faster and more accurate than other methods for Bayesian computation (32–36).

The cross-sectional study analyzed the associations between residence in areas with high environmental pressures and tumor characteristics using a series of mixed effects binary logistic regressions. Pathological/clinical staging (TNM III-IV; TNM IV), histological grading (grade 3), proliferation index (Ki-67 > 20%), hormonal receptors status (ER+ and/or PR+), epidermal receptor status (HER2/neu+), topography (C50.0–1), and laterality (bilateral) were considered outcome measures binary variables. For each regression model, records with missing values for the analyzed outcome were excluded. Residence in areas with high environmental pressures was included as a fixed effect binary variable. Age class and year were included as fixed effects multinominal variables. Month was included as a cubic b-spline with 12 degrees of freedom. Patient ID and municipality of residence were included as random effects multinominal variables (random intercepts). Bayesian binary logistic regression models were fitted with the INLA approach for latent Gaussian models, computing odds ratios (OR) and 95% credible intervals (CrI). An independent and identically distributed random distribution was chosen for patient ID and municipality of residence (33–35).

The longitudinal study analyzed the associations between residence in areas with high environmental pressures, tumor characteristics, and death, using a mixed effects Cox proportional hazard regression. Time axis was the difference in days between the day of cancer diagnosis and the last day of follow-up (event or right censoring). All-cause death was considered as the outcome measure binary variable (event). The proportional hazard assumption was verified through the analysis of plotted survival curves between the different levels of the variables. Residence in areas with high environmental pressures, pathological/clinical staging (TNM I, II, III, IV), histological grading (grade 1, 2, 3), proliferation index (Ki-67 ≤ 20%, 21–50, >50%), hormonal receptors status (ER- and PR-, ER+ and/or PR+), epidermal receptor status (HER2/neu-, HER2/neu+), topography (C50.0–1, C50.2–5, C50.6–8), and laterality (right, left, and bilateral) were included as fixed effects binary or multinominal variables. An “NA” (not available) category was created for the records with missing values for the analyzed exposures. Age class and year were included as fixed effects multinominal variables. Month was included as a cubic b-spline with 12 degrees of freedom. Patient ID, municipality of residence, and tumor morphology were included as random effects multinominal variables (random intercepts). Bayesian Cox regression models were fitted with the INLA approach for latent Gaussian models, computing hazard ratios (HR) and 95% credible intervals (CrI). An independent and identically distributed random distribution was chosen for patient ID, municipality of residence, and tumor morphology, while a random walk model of order two was chosen for the baseline hazard function (32–36).

Generalized Variance Inflation Factors (GVIF) were calculated to test the presence of multicollinearity in the data. Sensitivity analyses were performed by examining the extent to which the results were affected by changes in methods, models, variables, influential observations, and inclusion/exclusion criteria. Different combinations of included patients and variables were tested, and for the included variables, different collapsed categories, as well as changes in the type of estimated effects (fixed or random), were also tested. The models were iteratively refitted by excluding from the dataset each age class, year, and month one at a time.

## Results

Baseline patients and disease characteristics are shown in Table 1. A total of 10,445 person-years were observed, 4,068 for residents in SIN and 997 for deceased patients, with a median (IQR) age of 61.0 (50.0,72.0) years.

**Table 1:** Baseline patients and disease characteristics and follow-up survival status in the female breast cancer cohort by residence in SIN and survival status.

Baseline patients and disease characteristics and follow-up survival status	Female breast cancer cohort				
	*N* = 2,837; person-years = 10,445				
	Extra-SIN	SIN	Survived	Deceased	Total
*Age*					
Age [Median(IQR)]	61.0 (50.0;72.0)	61.0 (50.0;73.0)	59.0 (49.0;69.0)	76.0 (63.5;84.0)	61.0 (50.0;72.0)
30–39 [*n* (%)]	91 (5.32%)	47 (4.17%)	129 (5.40%)	9 (2.01%)	138 (4.86%)
40–49 [*n* (%)]	314 (18.36%)	213 (18.90%)	502 (21.00%)	25 (5.59%)	527 (18.58%)
50–59 [*n* (%)]	385 (22.51%)	269 (23.87%)	601 (25.15%)	53 (11.86%)	654 (23.05%)
60–69 [*n* (%)]	427 (24.97%)	239 (21.21%)	590 (24.69%)	76 (17.00%)	666 (23.48%)
70–79 [*n* (%)]	297 (17.37%)	212 (18.81%)	398 (16.65%)	111 (24.83%)	509 (17.94%)
≥80 [*n* (%)]	196 (11.46%)	147 (13.04%)	170 (7.11%)	173 (38.70%)	343 (12.09%)
*Year*					
2015 [*n* (%)]	337 (19.71%)	203 (18.01%)	418 (17.49%)	122 (27.29%)	540 (19.03%)
2016 [*n* (%)]	282 (16.49%)	190 (16.86%)	373 (15.61%)	99 (22.15%)	472 (16.64%)
2017 [*n* (%)]	260 (15.20%)	190 (16.86%)	374 (15.65%)	76 (17.00%)	450 (15.86%)
2018 [*n* (%)]	278 (16.26%)	169 (15.00%)	375 (15.69%)	72 (16.11%)	447 (15.76%)
2019 [*n* (%)]	288 (16.84%)	209 (18.54%)	451 (18.87%)	46 (10.29%)	497 (17.52%)
2020 [*n* (%)]	265 (15.50%)	166 (14.73%)	399 (16.69%)	32 (7.16%)	431 (15.19%)
*Pathological/clinical staging*					
TNM I [*n* (%)]	646 (37.78%)	421 (37.36%)	1,012 (42.34%)	55 (12.30%)	1,067 (37.61%)
TNM II [*n* (%)]	536 (31.35%)	335 (29.72%)	793 (33.18%)	78 (17.45%)	871 (30.70%)
TNM III [*n* (%)]	234 (13.68%)	148 (13.13%)	302 (12.64%)	80 (17.90%)	382 (13.46%)
TNM IV [*n* (%)]	113 (6.61%)	92 (8.16%)	69 (2.89%)	136 (30.43%)	205 (7.23%)
NA [*n* (%)]	181 (10.58%)	131 (11.62%)	214 (8.95%)	98 (21.92%)	312 (11.00%)
*Histological grading*					
Grade 1 [*n* (%)]	115 (6.73%)	70 (6.21%)	177 (7.41%)	8 (1.79%)	185 (6.52%)
Grade 2 [*n* (%)]	844 (49.36%)	561 (49.78%)	1,255 (52.51%)	150 (33.56%)	1,405 (49.52%)
Grade 3 [*n* (%)]	603 (35.26%)	387 (34.34%)	795 (33.26%)	195 (43.62%)	990 (34.90%)
NA [*n* (%)]	148 (8.65%)	109 (9.67%)	163 (6.82%)	94 (21.03%)	257 (9.06%)
*Proliferation index*					
Ki-67 ≤ 20% [*n* (%)]	1,059 (61.93%)	710 (63.00%)	1,569 (65.65%)	200 (44.74%)	1,769 (62.35%)
Ki-67 21–50% [*n* (%)]	410 (23.98%)	256 (22.72%)	548 (22.93%)	118 (26.40%)	666 (23.48%)
Ki-67 > 50% [*n* (%)]	168 (9.82%)	110 (9.76%)	213 (8.91%)	65 (14.54%)	278 (9.80%)
NA [*n* (%)]	73 (4.27%)	51 (4.53%)	60 (2.51%)	64 (14.32%)	124 (4.37%)
*Hormonal receptors status*					
ER- and PR- [*n* (%)]	217 (12.69%)	144 (12.78%)	280 (11.72%)	81 (18.12%)	361 (12.72%)
ER+ and/or PR+ [*n* (%)]	1,428 (83.51%)	934 (82.87%)	2,057 (86.07%)	305 (68.23%)	2,362 (83.26%)
NA [*n* (%)]	65 (3.80%)	49 (4.35%)	53 (2.22%)	61 (13.65%)	114 (4.02%)
*Epidermal receptor status*					
HER2/neu- [*n* (%)]	1,326 (77.54%)	867 (76.93%)	1,888 (79.00%)	305 (68.23%)	2,193 (77.30%)
HER2/neu+ [*n* (%)]	274 (16.02%)	176 (15.62%)	388 (16.23%)	62 (13.87%)	450 (15.86%)
NA [*n* (%)]	110 (6.43%)	84 (7.45%)	114 (4.77%)	80 (17.90%)	194 (6.84%)
*Topography*					
C50.0–1 [*n* (%)]	172 (10.06%)	101 (8.96%)	213 (8.91%)	60 (13.42%)	273 (9.62%)
C50.2–5 [*n* (%)]	1,047 (61.23%)	695 (61.67%)	1,526 (63.85%)	216 (48.32%)	1,742 (61.40%)
C50.6–8 [*n* (%)]	337 (19.71%)	210 (18.63%)	485 (20.29%)	62 (13.87%)	547 (19.28%)
NA [*n* (%)]	154 (9.01%)	121 (10.74%)	166 (6.95%)	109 (24.38%)	275 (9.69%)
*Laterality*					
Right [*n* (%)]	800 (46.78%)	549 (48.71%)	1,157 (48.41%)	192 (42.95%)	1,349 (47.55%)
Left [*n* (%)]	835 (48.83%)	531 (47.12%)	1,153 (48.24%)	213 (47.65%)	1,366 (48.15%)
Bilateral [*n* (%)]	36 (2.11%)	30 (2.66%)	47 (1.97%)	19 (4.25%)	66 (2.33%)
NA [*n* (%)]	39 (2.28%)	17 (1.51%)	33 (1.38%)	23 (5.15%)	56 (1.97%)
*Residence in SIN*					
Extra-SIN [*n* (%)]	1,710 (100.00%)	0 (0.00%)	1,460 (61.09%)	250 (55.93%)	1,710 (60.27%)
SIN [*n* (%)]	0 (0.00%)	1,127 (100.00%)	930 (38.91%)	197 (44.07%)	1,127 (39.73%)
*Survival status at the end of follow-up*					
Survived [*n* (%)]	1,460 (85.38%)	930 (82.52%)	2,390 (100.00%)	0 (0.00%)	2,390 (84.24%)
Deceased [*n* (%)]	250 (14.62%)	197 (17.48%)	0 (0.00%)	447 (100.00%)	447 (15.76%)
Days of follow-up [Median (IQR)]	1,324 (795;1,972)	1,280 (758;1,903)	1,421 (889;2,025)	695 (261;1,272)	1,312 (774;1,936)
Person-years [Sum]	6,377	4,068	9,448	997	10,445

Province of Taranto, 2015–2020, follow-up to 31/12/2021. N: cohort.

The results of the mixed effects Bayesian binary logistic regression models are reported in Table 2. Adjusting for baseline age class, year, month, patient ID, and municipality of residence, the fixed effect variable residence in SIN did not appear to be clearly associated with the prevalence of the investigated tumor characteristics. However, the lower limit of the 95% credible interval for TNM IV is quite close to one (OR 1.29, 95% CrI 0.96–1.73).

**Table 2:** Results of the mixed effects Bayesian INLA binary logistic regression models in the female breast cancer cohort, adjusted for baseline age class, year, month, patient ID, and municipality of residence.

Mixed effects INLA binary logistic regressions	Female breast cancer cohort							
	TNM III-IV		TNM IV		Grade 3		Ki-67 > 20%	
	*N* = 2,525; *n* = 587		*N* = 2,525; *n* = 205		*N* = 2,580; *n* = 990		*N* = 2,713; *n* = 944	
Fixed effect	OR	95% CrI	OR	95% CrI	OR	95% CrI	OR	95% CrI
*Residence in SIN*								
Extra-SIN	1.00	(ref)	1.00	(ref)	1.00	(ref)	1.00	(ref)
SIN	1.09	0.88–1.42	1.29	0.96–1.73	0.98	0.83–1.16	0.95	0.81–1.13

Mixed effects INLA binary logistic regressions	Female breast cancer cohort							
	ER+ and/or PR+		HER2/neu+		C50.0–1		Bilateral	
	*N* = 2,723; *n* = 2,362		*N* = 2,643; *n* = 450		*N* = 2,562; *n* = 273		*N* = 2,781; *n* = 66	
Fixed effect	OR	95% CrI	OR	95% CrI	OR	95% CrI	OR	95% CrI
*Residence in SIN*								
Extra-SIN	1.00	(ref)	1.00	(ref)	1.00	(ref)	1.00	(ref)
SIN	0.97	0.77–1.22	0.99	0.80–1.22	0.86	0.66–1.12	1.25	0.76–2.05

Province of Taranto, 2015–20, follow-up to 31/12/2021. Outcome (prevalence): tumor characteristic. N: prevalent cases and non-cases. *n*: prevalent cases.

**Figure 2:**
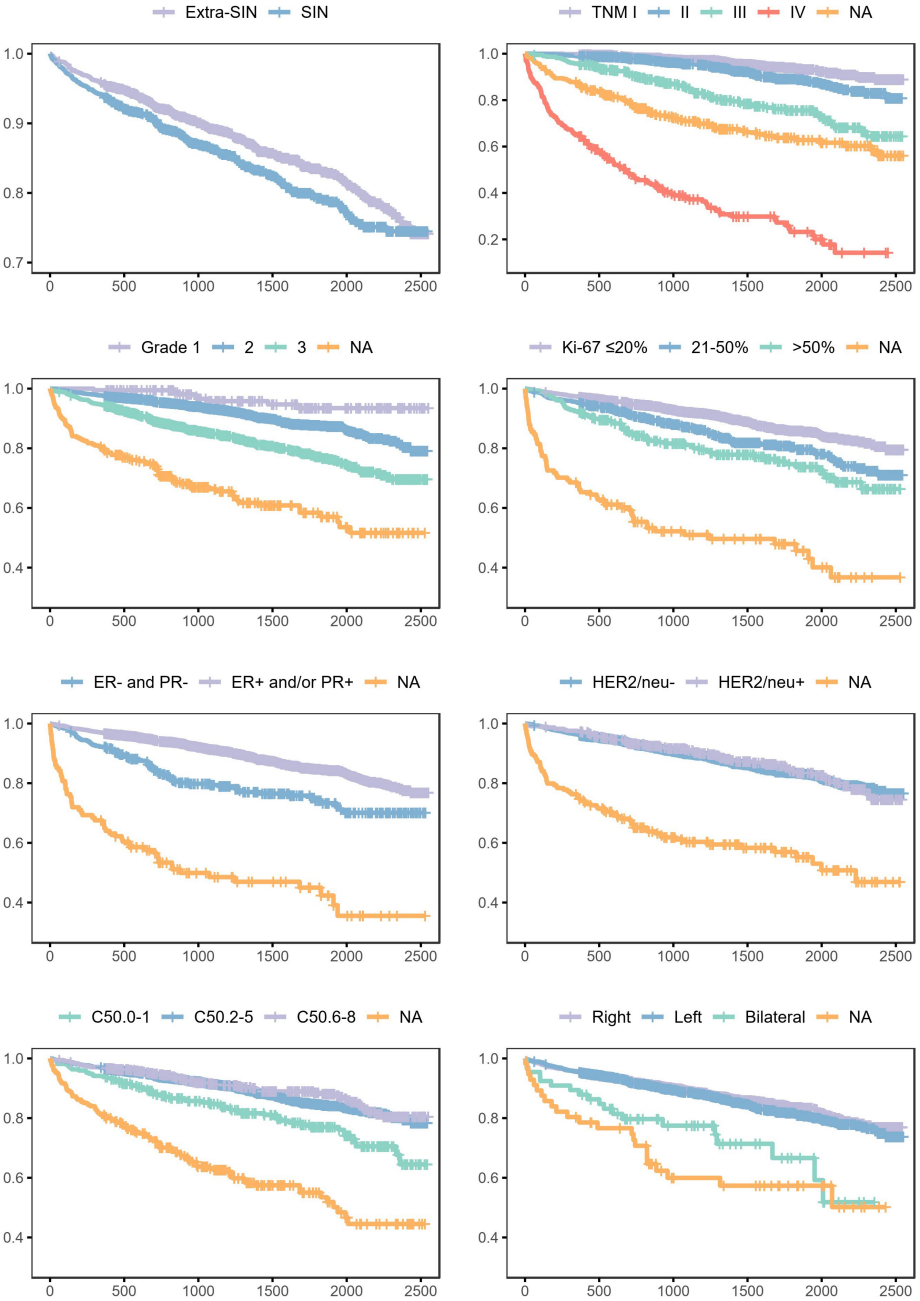
Survival probabilities in the female breast cancer cohort, conditional on each analyzed variable and unconditional on other variables. Province of Taranto, 2015–2020, follow-up to 31/12/2021. Time: days of follow-up. Outcome (incidence): all-cause death.

Survival probabilities conditional on each analyzed variable and unconditional on other variables are shown in Figure 2. The curves suggested unconditional associations between survival probability and residence in SIN, pathological/clinical staging, histological grading, proliferation index, hormonal receptors status, epidermal receptor status, topography, and laterality. The results of the mixed effects Bayesian Cox proportional hazard regression model are reported in Table 3. Mutually adjusting and adjusting for baseline age class, year, month, patient ID, municipality of residence, and tumor morphology, the fixed effects variables associated with a higher death rate were residence in SIN (HR 1.22, 95% CrI 1.01–1.48), TNM III (HR 2.77, 95% CrI 1.93–3.97), IV (HR 17.05, 95% CrI 11.94–24.34) and NA (HR 4.13, 95% CrI 2.87–5.95), grade 3 (HR 2.50, 95% CrI 1.20–5.23) and NA (HR 2.18, 95% CrI 1.02–4.66), Ki-67 21–50% (HR 1.42, 95% CrI 1.10–1.83) and > 50% (HR 1.81, 95% CrI 1.29–2.55), and bilateral localization (HR 1.65, 95% CrI 1.01–2.68). Mutually adjusting and adjusting for baseline age class, year, month, patient ID, municipality of residence, and tumor morphology, the fixed effects variables associated with lower death rate were ER+ and/or PR+ (HR 0.61, 95% CrI 0.45–0.81) and HER2/neu + (HR 0.59, 95% CrI 0.44–0.79).

**Table 3:** Results of the mixed effects Bayesian INLA Cox proportional hazard regression model in the female breast cancer cohort, mutually adjusted and adjusted for baseline age class, year, month, patient ID, municipality of residence, and tumor morphology.

Mixed effects INLA Cox proportional hazard regression	Female breast cancer cohort	
	All-cause death	
	*N* = 2,837; person-years = 10,445; *n* = 447	
Fixed effects	HR	95% CrI
*Residence in SIN*		
Extra-SIN	1.00	(ref)
SIN	1.22	1.01–1.48
*Pathological/clinical staging*		
TNM I	1.00	(ref)
TNM II	1.26	0.88–1.79
TNM III	2.77	1.93–3.97
TNM IV	17.05	11.94–24.34
NA	4.13	2.87–5.95
*Histological grading*		
Grade 1	1.00	(ref)
Grade 2	1.93	0.94–3.96
Grade 3	2.50	1.20–5.23
NA	2.18	1.02–4.66
*Proliferation index*		
Ki-67 ≤ 20%	1.00	(ref)
Ki-67 21–50%	1.42	1.10–1.83
Ki-67 > 50%	1.81	1.29–2.55
NA	1.09	0.41–2.89
*Hormonal receptors status*		
ER- and PR-	1.00	(ref)
ER+ and/or PR+	0.61	0.45–0.81
NA	0.88	0.33–2.30
*Epidermal receptor status*		
HER2/neu-	1.00	(ref)
HER2/neu+	0.59	0.44–0.79
NA	1.40	0.87–2.23
*Topography*		
C50.0–1	1.00	(ref)
C50.2–5	0.88	0.65–1.18
C50.6–8	0.81	0.56–1.17
NA	0.95	0.67–1.34
*Laterality*		
Right	1.00	(ref)
Left	1.03	0.84–1.26
Bilateral	1.65	1.01–2.68
NA	1.28	0.80–2.03

Province of Taranto, 2015–2020, follow-up to 31/12/2021. Time: days of follow-up. Outcome (incidence): all-cause death. N: incident cases and non-cases. n: incident cases.

## Discussion

The results of the present study confirmed that TNM staging, histological grading, proliferation index, estrogen and/or progesterone positivity, HER2/neu positivity, and bilaterality are independent prognostic factors in breast cancer patients. In our cohort, only topography did not seem to be independently associated with the analyzed outcome. Of interest was the finding during the follow-up period of an overall average negative association between HER2/neu positivity and death rate in our cohort, although it may be non-constant over time. This association could be explained by the development and implementation of HER2/neu targeting treatments. In fact, patients with HER2/neu + tumors are amenable to treatment with targeted biological agents such as trastuzumab. For these reasons, the presence of this marker could be assumed as a proxy of treatment with these drugs, which is information not directly available in the cancer registry. Given this assumption, our findings could be supported by the scientific literature about the improvement of survival in the treated patients (1, 3). Another interesting result was the negative prognostic value of the presence of missing data for some variables (“NA” category) in our cohort. This could be partly explained by the fact that patients without information on some variables (e.g., grading) could correspond to poor prognosis patients who, due to a severe condition at the time of diagnosis, were unable to undergo further interventions or investigations (37).

Regarding the impacts of environmental pressures on tumor characteristics, this study found no clear association between living in the SIN and the prevalence of the prognostic factors mentioned above. Conversely, the most important finding seems to be the association between residence in SIN and increased all-cause death rate. This result was observed independently of all other factors analyzed, as the HR was adjusted for the other variables included in the Bayesian mixed effects regression model. In summary, this suggests that the patients in the studied cohort that resided in the SIN have an adjusted excess relative risk for all-cause mortality of 22% (95% CrI 1–48%) compared to the residents in the other municipalities of the province.

The specific environmental factors that may be associated with this excess mortality could be related to some documented anthropic pressures in the SIN: harbor, discharge, oil refinery, and steel plant (29, 30). Several pieces of evidence in the study area have shown the contamination of environmental, feed, and food matrices (e.g., mussels and eggs) with metals and persistent organic pollutants, such as dioxins and PCBs, in relation to potential foodborne exposure. Some of these substances or their metabolites/markers have also been detected in human biological samples (17, 20–25). With regard to air pollution, studies have documented air pollution originating from the industrial area (e.g., particulate matter, sulfur dioxide, and polycyclic aromatic hydrocarbons) and the impacts on human health (16, 18, 19, 26, 28–30, 38).

Our findings confirm previous knowledge regarding the increased risk of all-cause mortality reported for women who resided in the SIN of Taranto (16, 19, 26, 29, 30). However, they also suggest that the excess relative risk in the cohort analyzed may be greater. In fact, recent epidemiological studies on all the female population residing in the area have shown an excess relative risk for all-cause mortality of 7% (90% CI 5–9%) in the SIN of Taranto compared to the Apulia Region in the years 2013–17 (29). Although we are considering different periods and methods, and the credible interval of our study fully contains the aforementioned estimate with its confidence interval, we could not discount the possibility that the excess mortality risk in the SIN of Taranto might be higher in the cohort of women with breast cancer. Therefore, our findings suggest that frail female breast cancer patients may be more vulnerable to the risks associated with a disadvantaged or polluted external environment. This could be also consistent with the findings reported in the few studies that analyzed the association between socio-economic-environmental pressures and the prognosis of female breast cancer (12, 14, 15).

These results raise possible ethical questions, if confirmed. As a matter of fact, several epidemiological studies have reported an increased risk for breast cancer incidence and hospitalization and for all-cause mortality in the entire female population that resided in the SIN of Taranto (16, 19, 26, 27, 29, 30). According to the present study, this excess relative risk for all-cause mortality might be higher in the cohort of female breast cancer patients.

However, since this study used an ecological variable (i.e., residence in the SIN of Taranto) to ascertain exposure to environmental pressures, this approach is potentially prone to ecological fallacy. In addition, there is a lack of specificity in the exposure assessment as the specific chemical pollutants could be varied and come from different sources in the studied region (37). Another limitation of the study could be the lack of information about genetics and hereditary. These data are unfortunately not available in cancer registries or health records. However, part of the influence of these factors may have been indirectly captured in the analysis using mixed models with random effects, which take into account the heterogeneity between patients and areas.

Nonetheless, regardless of these limitations, all the other evidence already available on Taranto gives a high *a priori* plausibility of the association between residence in SIN and increased mortality detected in this study, also considering that this association persists despite adjustment for all other measured patients and disease characteristics. Moreover, in addition to the well-known pressures of a strictly environmental nature, the two municipalities of Taranto and Statte present relatively high municipality-level deprivation indexes. This is a regionally referenced deprivation index that used the individual data of the general population and housing census of 2011. For the calculation of the index, five conditions were chosen by the authors to best describe the multidimensional concept of social and material deprivation: low level of education, being unemployed, living on rent, living on rent, and living in a single-parent family. The index was calculated as the sum of standardized indicators and is also available and categorized into quintiles (39). Although including the municipality of residence as a random effect in the regression models provided some ecological-level adjustment for the deprivation index, it can also be useful to consider the value of the index itself for descriptive purposes when interpreting the results. Anyhow, particular attention should be paid to the interpretation of this index both due to its ecological-level indicator nature and since the latest available index relates to the 2011 census (39). Therefore, the available deprivation index cannot be guaranteed to accurately indicate individual-level deprivation during the years covered by the present study.

However, it is important to consider that socio-economic status, deprivation, and inequalities could not only exert an effect on lifestyle harmful habits (e.g., cigarette smoking), health conditions, and mortality but also potentially affect the utilization of health services (39, 40). Furthermore, in this regard, the SIN corresponds almost completely to the provincial capital Taranto, which could potentially influence access to health services at a territorial and hospital level, and in terms of regional and extra-regional mobility. This is linked to another limitation of the study, which is the lack of available data about the diagnostic-therapeutic pathways followed by these patients, including information regarding their access to breast cancer screening services. On the other hand, the fact that both SIN and extra-SIN municipalities belong to the same Local Health Authority, and so consequentially the entire studied cohort could virtually access the same healthcare and screening services, did not lead us to suppose that there could be relevant biases in relation to these aspects (37).

To summarize, as mentioned previously, the lack of information about individual-level environmental exposures, genetics and hereditary factors, socio-cultural indicators, harmful habits, and utilization of healthcare services could represent a limitation of the present study. However, from another perspective, the same elements could also be considered starting points for what can be done in the future. Specifically, it would be interesting to update and expand upon this epidemiologic study by recovering further individual-level data about genetics and hereditary factors (BRCA1, BRCA2, PTEN, and TP53 mutations) (41), environmental exposures (distance from polluting sources, exposure to airborne pollutants through dispersion models, and biomonitoring), socio-economic factors (updated indicators of deprivation at individual or census-tract level), access to secondary prevention programs (screening path through mammography, echography, and genetic tests), and diagnostic-therapeutic-surgical paths (timing, place, and type of interventions).

In conclusion, the results confirmed the independent prognostic values of different female breast cancer characteristics. Despite the limitations discussed above, even after adjusting for patients and disease characteristics, in the cohort of women with invasive breast cancer, residence in the SIN of Taranto appeared to be associated with an increased death rate.

## Data availability statement

The original contributions presented in the study are included in the article/supplementary materials, further inquiries can be directed to the corresponding author.

## Ethics statement

Ethical approval was not required for the study involving humans in accordance with the local legislation and institutional requirements. Written informed consent to participate in this study was not required from the participants or the participants’ legal guardians/next of kin in accordance with the national legislation and the institutional requirements.

## Author contributions

OVG: Conceptualization, Data curation, Formal analysis, Investigation, Methodology, Project administration, Supervision, Validation, Visualization, Writing – original draft, Writing – review & editing. SC: Conceptualization, Data curation, Investigation, Methodology, Supervision, Validation, Writing – review & editing. MT: Data curation, Writing – review & editing. CG: Data curation, Writing – review & editing. AB: Data curation, Writing – review & editing. GML: Data curation, Writing – review & editing. IR: Data curation, Software, Writing – review & editing. LB: Data curation, Resources, Writing – review & editing. RS: Funding acquisition, Methodology, Validation, Writing – review & editing. FA: Investigation, Validation, Writing – review & editing. SM: Data curation, Funding acquisition, Project administration, Resources, Supervision, Writing – review & editing. AM: Data curation, Funding acquisition, Project administration, Resources, Supervision, Writing – review & editing.

## Glossary

AIRTUM: Italian Association of Cancer Registries
BRCA1: Breast Cancer gene 1
BRCA2: Breast Cancer gene 2
CI: Confidence Interval
CrI: Credible Interval
DALY: Disability-Adjusted Life Year
ER: Estrogen Receptor
FISH: Fluorescence *In Situ* Hybridization
GVIF: Generalized Variance Inflation Factor
HER2: Human Epidermal Growth Factor Receptor 2
HR: Hazard Ratio
ICD: International Classification of Disease
ICDO: International Classification of Disease for Oncology
ID: Identifier
IHC: Immunohistochemistry
INLA: Integrated Nested Laplace Approximation
IQR: Interquartile Range
NA: Not Available
OR: Odds Ratio
PCB: Polychlorinated Byphenyl
PR: Progesterone Receptor
PTEN: Phosphatase and Tensin Homolog
SIN: Contaminated Site of National Interest
TNM: Tumor, Node, Metastases
TP53: Tumor Protein 53
