# Corrigendum: Environmental pressures, tumor characteristics, and death rate in a female breast cancer cohort: a seven-years Bayesian survival analysis using cancer registry data from a contaminated area in Italy

**DOI:** 10.3389/fpubh.2024.1441736

**Published:** 2024-07-31

**Authors:** Orazio Valerio Giannico, Simona Carone, Margherita Tanzarella, Claudia Galluzzo, Antonella Bruni, Giovanna Maria Lagravinese, Ivan Rashid, Lucia Bisceglia, Rodolfo Sardone, Francesco Addabbo, Sante Minerba, Antonia Mincuzzi

**Affiliations:** ^1^Unit of Statistics and Epidemiology, Local Health Authority of Taranto, Taranto, Italy; ^2^Coordination Center of the Apulia Cancer Registry, Strategic Regional Agency for Health and Social Care of Apulia, Bari, Italy; ^3^Healthcare Management, Local Health Authority of Taranto, Taranto, Italy

**Keywords:** breast cancer, female breast cancer, cancer survival, environmental contamination, environmental pollution, cancer epidemiology

In the published article, there was an error in Table 1 as published. The age class “50-59” has been erroneously reported as “59-59” due to a typo. The correct label is “50-59”.

The authors apologize for this error and state that this does not change the scientific conclusions of the article in any way. The original article has been updated.

